# Aerogel-Based Phase Change Materials Meet Flame Retardancy: From Materials to Properties

**DOI:** 10.3390/gels11110923

**Published:** 2025-11-19

**Authors:** Panpan Zhao, Shudi Ying, Riming Hu, Jiachen Ma, Xuchuan Jiang

**Affiliations:** 1 Institute for Smart Materials & Engineering, University of Jinan, Jinan 250022, China; 2 Department of Molecular Chemistry and Materials Sciences, Weizmann Institute of Science, Rehovot 76100, Israel; 3 School of Intelligent Manufacturing, Huzhou College, Huzhou 313000, China

**Keywords:** aerogels, phase change materials, flame retardancy, energy storage/release, latent heat

## Abstract

Energy storage materials play a crucial role in enhancing system efficiency by bridging the mismatch between energy supply and demand. Among them, organic phase change materials (PCMs) are particularly attractive due to their high energy storage density, no phase segregation and ability to maintain nearly constant temperatures during phase transitions. However, their practical application is hindered by drawbacks such as leakage and flammability. Aerogels, characterized by high porosity, low density, and tunable structures, provide effective support matrices for encapsulating PCMs, thereby improving shape stability and enabling fire safety improvements when combined with flame-retardant strategies. Despite significant progress in PCM and aerogel research over the past decade, comprehensive studies dedicated to flame-retardant aerogel-based PCMs remain limited. This review systematically summarizes current flame-retardant approaches for aerogel-based PCMs, highlights recent advances in aerogel-supported systems, and outlines the key challenges and future opportunities for developing next-generation energy storage composites with improved thermal reliability, safety, and sustainability.

## 1. Introduction

Phase change materials (PCMs) are substances that undergo reversible phase transitions—typically between an amorphous and one or more crystalline states—within a defined temperature range [1]. By storing and releasing significant amounts of latent heat during these transitions, PCMs enable efficient thermal energy management and enhanced solar energy utilization. Consequently, they find widespread applications in areas such as smart textiles [2], building materials [3,4], and battery [5] technologies. Organic solid—liquid PCMs are favored for their high thermal storage density, low cost and minimal supercooling, making them ideal for various applications. Examples of such PCMs include aliphatics, polyols, polyhydric alcohols, and fatty acids [6]. However, large-scale deployment of this technology requires overcoming key research and development challenges, including leakage during solid–liquid transition, low thermal stability, and poor flame retardant properties [7,8].

Encapsulating PCMs within three-dimensional (3D) crosslinked networks is among the most effective strategies for preventing leakage during phase transition and, in some cases, for enhancing flame retardancy and thermal stability. Among the materials explored for this purpose, aerogels have attracted significant attention due to their unique properties, including high porosity, light weight, and a high specific surface area [9]. Numerous reviews have summarized the development of aerogels across both inorganic and organic categories. Inorganic aerogels include well-studied systems such as silica aerogels [10] and carbon aerogels [11], while organic aerogels encompass materials such as phenolic aerogels [12], nanocellulose-based aerogels [13], and wood aerogels [14]. By encapsulating PCMs within their porous network, aerogels effectively prevent leakage, enhance mechanical stability, and improve thermal insulation.

The intrinsic properties of the supporting aerogels play a decisive role: inorganic aerogels generally provide inherent fire resistance but are fragile, whereas organic aerogels, despite their structural advantages, are often flammable [15]. This contrast underscores the importance of tailoring the choice or modification of aerogel matrices to achieve both high energy storage efficiency and enhanced flame-retardant performance in composite PCMs. Moreover, given the intrinsic flammability of most organic PCMs, integrating flame-retardant strategies into aerogel-based PCMs is essential to ensure safe and sustainable operation. Conventional approaches include physical blending of flame-retardant additives [16], intrinsic incorporation of flame-retardant groups [17,18], and surface coatings [19]. These strategies function through gas-phase radical quenching, condensed-phase char formation, or synergistic effects, thereby reducing heat release, suppressing flame propagation, and enhancing thermal stability.

Recent reviews have addressed various aspects of PCMs, including their thermal performance [20], encapsulation strategies [21,22], thermo-physical properties [23], and composite formulations and fabrication techniques [24]. Although literature exists on flame-retardant PCMs [25,26] and on flame-retardant aerogels [15], the integration of flame retardancy into aerogel-based PCMs has received limited systematic attention. Most prior works either overlook fire safety considerations or address them in a fragmented manner without establishing clear structure–function relationships. A comprehensive review that systematically classifies the materials and elucidates the flame retardancy mechanisms of aerogel-based PCMs within a unified framework is presently lacking. This review fills that gap by offering a focused and structured synthesis of the recent progress in flame-retardant aerogel-based PCMs. Specifically, we classify current systems based on the type of aerogel matrix (organic, inorganic, or hybrid) and the flame-retardant strategies employed (barrier effect, endothermic action, radical trapping, etc.). This review provides an in-depth overview of flame-retardant strategies applicable to aerogel-based PCMs and elucidates the relationships between material composition, structure, and fire safety. Furthermore, it highlights key challenges and research gaps, offering perspectives to guide the development of next-generation aerogel-based PCMs with high thermal energy storage efficiency, enhanced fire safety, and improved environmental sustainability.

## 2. Flame-Retardant Methods

**Figure 1:**
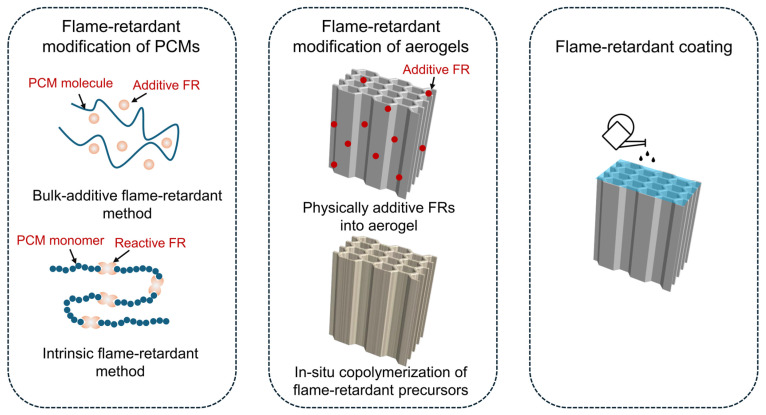
Schematic of traditional flame-retardant methods of composite PCMs.

Flame retardants (FRs) are compounds incorporated into organic polymers or plastics to inhibit ignition and suppress fire propagation. Previous reviews have summarized the major classes of conventional FRs and their underlying mechanisms, which typically operate through gas-phase or condensed-phase pathways [25,27,28]. Gas-phase flame retardancy primarily relies on the dilution of reactive species and radical scavenging—for example, through phosphorus-derived radicals (e.g., PO·, PO_2_·, HPO_2_·) that quench chain reactions. In contrast, condensed-phase mechanisms focus on physical barrier formation and catalytic carbonization/cross-linking, which suppress heat feedback and fuel release. Such protective layers can originate from the decomposition of flame-retardant polymers containing S, P, or Si, or from thermally stable inorganic fillers like MXene, BN, and clay [29]. In the context of PCMs, the primary flame-retardant approaches can be categorized into physical blending, intrinsic flame-retardant design, and flame-retardant coatings (Figure 1). This review will primarily focus on the first two strategies.

### 2.1. Physically Blending Method

Physically blending FRs, including organic and inorganic FRs, into PCMs represents the simplest strategy for enhancing fire safety. Organic phosphorus-containing FRs can promote char forming of materials and/or eliminate flame radicals in the gas phase during combustion [30]. Alkhazaleh et al. [31] developed a form-stable composite PCM (LA_RDP/EP) comprising 90 wt% lauric acid (LA) as the phase change material and 10 wt% resorcinol bis(diphenyl phosphate) (RDP) as the flame retardant within expanded perlite (EP). The resulting composite LA_RDP/EP exhibits an appropriate phase-transition temperature of 40–45 °C and a fusion latent heat of 86.02–88.29 J g^−1^, while significantly reducing the peak heat release rate (PHRR) and total heat release (THR). These improvements in flame retardancy were attributed to the synergistic effect of EP’s inherent fire resistance and RDP’s radical-scavenging action during combustion.

Inorganic FRs—including metal hydroxides; inorganic P-, N-, and Si-based species; and boron compounds—are typically incorporated into polymers as physically dispersed additives. Their flame-retardant action arises from chemical and/or physical processes operating in the gas and/or condensed phases [32]. Among them, ammonium polyphosphate (APP), a halogen-free FR, is widely used and highly effective due to its condensed-phase intumescent action [33]. For instance, composite PCMs containing paraffin (PA), expanded graphite (EG), APP, red phosphorus (RP), and epoxy resin (ER) have been developed to improve both stability and flame resistance in battery modules [34]. In one case, the limiting oxygen index (LOI) of a PCM with an APP/RP mass ratio of 23/10 increased to 27.6%; meanwhile, the UL-94 test achieved a V-0 rating. The synergistic effect between APP and RP produces a dense carbonized interfacial layer that effectively shields the underlying matrix from oxygen and heat, thereby suppressing dripping and ensuring a robust flame-retardant effect. To further enhance performance, phosphorus/nitrogen (P/N) synergistic flame-retardant systems have been introduced. Li et al. [16] introduced a P/N synergistic flame retardant, composed of a triazine-based char-forming agent (CFA) and APP, into eutectic PA/polypropylene (PP) composites via physical blending for building applications. The PHRR of the resulting flame-retarded PCM (PCM6) was significantly reduced to 135.9 kW·m^−2^ compared to 1189.7 kW·m^−2^ for the PA/PP composite. The flame retardancy arises from the synergistic P/N flame-retardant effect, which increases the molten viscosity and promotes the formation of a stable char layer during combustion. The resulting char layer effectively insulated the matrix from heat and oxygen. A flame-retardant solid–solid PCM was developed by integrating octadecyl acrylate crosslinked networks with PA/EG, and an intumescent system of APP/melamine pyrophosphate (MPP)/dipentaerythritol [35]. The composite PCM achieved a UL-94 V-0 rating and exhibited densified char morphologies after combustion. The outstanding fire resistance results from synergistic flame-retardant mechanisms, including: (1) condensed-phase action through reinforced char layer formation; (2) gas-phase radical scavenging to inhibit combustion reactions; and (3) generation of thermally stable, ceramic-like barrier layers that suppress heat and mass transfer.

Metal hydroxides are low-cost, non-toxic, and thermally stable FRs that do not release toxic gases at elevated temperatures. They reduce smoke generation during plastic combustion and, via thermal decomposition, release water that reduces the temperature of the polymer matrix while diluting combustible gases and oxygen in the flame zone [36,37]. Magnesium chloride hexahydrate (MCH, MgCl_2_·6H_2_O) was incorporated as a flame-retardant and heat-absorbing component into a PA/EG/ER composite with ER as the supporting matrix to generate flame-retardant composite PCM (CPCM). The resulting CPCM achieved a UL-94 V-0 rating without any dripping of the sample and exhibited ultrahigh heat absorption (~800 J g^−1^) over 100–300 °C [38]. During combustion, MCH decomposes and releases H_2_O and HCl gases, which dilute the concentration of O_2_ near the CPCM surface, thereby contributing to gas-phase flame retardancy. Simultaneously, the resulting MgO can interact with EG to form a dense carbonaceous layer, which acts as a barrier to further oxygen penetration, thus enhancing condensed-phase flame retardancy. In addition, MCH absorbs a substantial amount of heat through its endothermic decomposition.

Silicon dioxide (SiO_2_), a fire-resistant inorganic porous material, is commonly used as a synergistic flame-retardant with APP and EG [39,40]. Liu et al. [41] prepared flame-retardant composite PCM using non-toxic silica-based flame retardant (SiO_2_ sol) as the flame retardant, EG as the shaping support, and PA as the PCM, which reduced volatile combustion products, including asphyxiating gases such as CO_2_ and CO. Table 1 summarizes the typical flame-retardant PCMs with phase change enthalpy and flame-retardant parameters. In general, increasing the content of FR additives leads to a significant reduction in PHRR and THR, confirming their effectiveness in suppressing combustion intensity and heat propagation. For instance, composites with 15–45 wt% FR loading commonly exhibit PHRR reductions of 30–60% relative to neat PCM systems. Moreover, the LOI typically increases proportionally with FR content, often exceeding 28%, indicating improved flame retardancy. However, this improvement in fire safety is frequently accompanied by a reduction in Δ*H*_m_. As shown in Table 1, increasing FR content beyond 20 wt% can result in a 10–40% decrease in Δ*H*_m_, primarily due to the dilution of the active PCM phase and disruption of its crystallinity. The extent of this trade-off depends strongly on the morphological compatibility and dispersion of the FR additive within the matrix. For example, well-dispersed nano-additives or intrinsically flame-retardant frameworks (e.g., EG) can partially mitigate the loss in latent heat by reducing the addition of FRs. Composite PCMs incorporating intumescent flame retardants (IFRs)—comprising an acid source, a carbonizing source, and a foaming or blowing source [42]—have been developed to enhance flame resistance. These composite PCMs can maintain Δ*H*_m_ values above 80 J g^−1^ while achieving LOI values above 30%. Among, APP is the most widely used acid source, as it decomposed at a higher temperature to generate polyphosphoric acid [33]. The acid can dehydrate and carbonize polymers to form char layers, which can inhibit oxygen and heat transfer, thus achieving improved flame retardancy. These trends underscore the importance of carefully tuning FR loading, additive morphology, and interfacial compatibility to achieve synergistic optimization of thermal energy storage and flame retardancy.

In addition, the integration of FR additives—such as metal hydroxides, P-based compounds, or SiO_2_—into polymer matrices not only enhances fire resistance but also significantly affects the viscoelastic behavior and mechanical stability of the resulting composites. Some additives can act as reinforcing fillers, restricting polymer chain mobility and increasing the stiffness and storage modulus of the composite [43]. These changes in viscoelastic properties are particularly relevant in PCM systems, where cyclic thermal storage/release during melting and solidification may induce mechanical fatigue or dimensional instability. A more elastic or ductile matrix may accommodate thermal stresses, while a stiffer matrix can better maintain shape integrity but risks brittleness over time. The presence of FR additives can thus modulate the balance between elasticity and rigidity, influencing both thermal performance and structural durability. Moreover, some FRs contribute to improved thermal stability and anti-dripping property under fire, which correlates with enhanced viscoelastic stability at elevated temperatures [44,45]. Therefore, the design of PCM composites incorporating FR additives must take into account not only their flame-retardant performance but also their impact on the viscoelastic and mechanical behavior of the polymeric matrix.

**Table 1:** Key parameters of phase-change performance and flame retardancy in recently reported PCMs using additive flame retardants.

Supporting Matrix	PCMs	FRs	FR Loading (wt%)	Δ*H*_m_ (J g^−1^)	PHRR	THR	LOI (%)	Ref.
Expanded perlite	Lauric acid	Diphenyl phosphate	20	119.7	784 kW m^−2^	15.89 MJ m^−2^	-	[31]
ER/EG	PA	APP/red phosphorus (RP)	33	81.2	313.1 kW m^−2^	89.3 MJ m^−2^	27.6	[34]
PP	PA	Triazine char forming agent (CFA)/APP	30	119.2	135.9 kW m^−2^	68.3 MJ m^−2^	32.8	[16]
EG	PA	APP/melamine pyrophosphate (MPP)/dipentaerythritol	25	80.7	-	-	38.3	[35]
ER/EG	PA	Magnesium chloride hexahydrate (MCH)	44	-	-	107.5 MJ m^−2^	-	[38]
EG	n-Hexadecane	Silicon dioxide (SiO_2_)	-	145.6	-	-	-	[40]
Olefin block copolymer (OBC)	PA	Chopped carbon fiber (CF)/APP/EG	18	131.6	181.8 kW m^−2^	37.5 MJ m^−2^	37.5	[46]
OBC	PA	Ti_3_C_2_T_x_ MXene	20	168.8	620 W g^−1^	49.6 kJ g^−1^	-	[47]
SEPS (YH-4051)	PA	Aluminum hydroxide (ATH)/magnesium hydroxide (MTH)/APP	20	145.1	615.3 kW m^−2^	<120 MJ m^−2^	23.2	[48]
EG	PA	APP/pentaerythritol (PER)/melamine (MA)	-	-	448.0 kW m^−2^	114.2 MJ m^−2^	29.8	[49]
EG/N,N’-Methylenebisacrylamide (MBA)	Polyethylene glycol (PEG)	Microcapsule-coated ammonium polyphosphate (MFAPP)	19	76.34	452.23 kW m^−2^	60.09 MJ m^−2^	32.6	[50]
Styrene-maleic anhydride copolymer (SMA)	PEG	BP/EG	15	92.2	596.1 kW m^−2^	80.0 MJ m^−2^	-	[51]
Styrene–butadiene–styrene (SBS)/EG	PA	Methylphenyl silicone (MPS)/triphenyl phosphate (TPP)	24	134.0	486.5 kW m^−2^	110 MJ m^−2^	28.3	[52]
MOF/EG/ER	Polyamide	MOF/APP/EG	33	60.59	119.33 kW m^−2^	39.06 MJ m^−2^	31.3	[53]
EG/1,6-hexanediol diacrylate (HDDA)	Octadecyl acrylate (OA)	Al(OH)_3_	15	71.53	204.4 kW m^−2^	181.6 MJ m^−2^	-	[54]
Poly (glycerol-itaconic acid)	PEG	APP	15	70.11	413 kW m^−2^	77.3 MJ m^−2^	28.7	[55]
MXene	P-modified stearyl alcohol (SAL)	-		120.1	440.2 kW m^−2^	61.4 MJ m^−2^	-	[56]

Note: Units are specified individually to reflect the original measurement technique. kW·m^−2^ values are obtained from cone calorimeter tests, while W·g^−1^ values are from microscale combustion calorimetry (MCC).

### 2.2. Intrinsic Flame-Retardant Methods

Although the bulk-additive flame-retardant strategies have shown promising effects in enhancing the flame retardancy of PCMs, certain limitations remain. These include FR migration, poor compatibility, and deterioration of PCMs’ intrinsic properties, such as reduced phase change enthalpy, all of which require urgent attention. To overcome these limitations, intrinsic approaches that chemically incorporate flame-retardant structures into the molecular chains of PCMs are considered more effective. In this section, we will discuss the conventional intrinsic flame-retardant methods that have been widely applied over the past few decades.

#### 2.2.1. Ionic Liquid-Based PCMs

**Figure 2:**
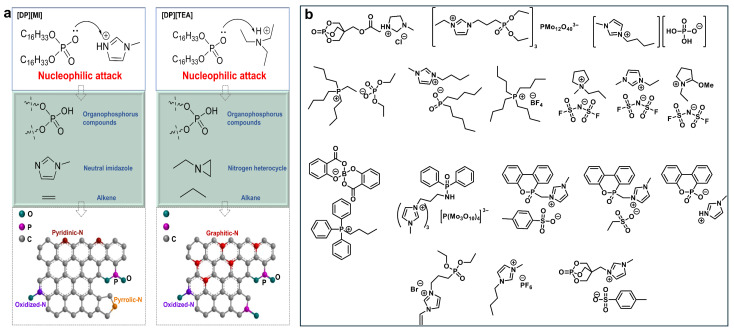
(**a**) Flame-retardant mechanism of [DP][MI] and [DP][TEA]; Reproduced with permission [64]. (**b**) Structures of IL-based flame retardants [61,65,66,67,68,69,70,71,72,73,74,75,76,77,78].

Ionic liquids (ILs), defined as compounds composed entirely of ions with melting points below 100 °C, often exhibit “green” and “designer” properties to a useful degree [57]. Zhu et al. [58] investigated the thermodynamic properties of a series of imidazolium-based ionic liquids or ethylamine tetrafluoroborates, reporting heats of fusion ranging from 37.6 to 152.6 J g^−1^. However, the fire safety of these ionic liquid-based PCMs was not examined. Generally, the thermal degradation mechanism of ILs mainly involves reverse Menshutkin reactions and Hofmann eliminations, yielding terminal alkenes and protonated anions [59,60]. In particular, when designed with phosphorus and/or nitrogen, they can enhance the fire safety of materials through promoting char formation and/or diluting flammable gases [61,62,63]. Two novel P/N-containing ILs, [DP][MI] and [DP][TEA]—comprising imidazole (MI) or triethylamine (TEA) cations and a dicetyl phosphate (DP) anion—were synthesized for use in flame-retardant PCMs [64]. They exhibited high thermal enthalpies of 99.0 and 94.7 J g^−1^, respectively, and demonstrated rapid self-extinguishing behavior upon ignition. During thermal decomposition, [DP][MI] forms organophosphorus compounds through proton abstraction from the imidazole ring and alkyl chain cleavage, with stable imidazole structures persisting up to 600 °C. This leads to a P-N char residue formed via thermal rearrangement. In contrast, [DP][TEA] decomposes via intramolecular rearrangement and ethane loss, yielding nitrogen heterocycles. Thus, [DP][MI] showed superior fire resistance, attributed to the high char-forming capability of the MI cation, while the DP anion contributed to flame inhibition through both gas- and condensed-phase mechanisms (Figure 2a). To date, only limited research has explored ILs as flame-retardant PCMs; therefore, we summarize existing studies on IL-based flame retardants in Figure 2b to provide guidance for designing high-performance IL-based flame-retardant PCMs. ILs offer high structural tunability and chemical diversity, allowing flame-retardant elements such as P, N, B, and halogens to be incorporated into either the anion or cation [61,65,66,67,68,69,70,71,72,73,74,75,76,77,78]. These functionalized ILs have demonstrated excellent flame-retardant performance when applied in combustible material systems.

#### 2.2.2. PU-Based PCMs

**Figure 3:**
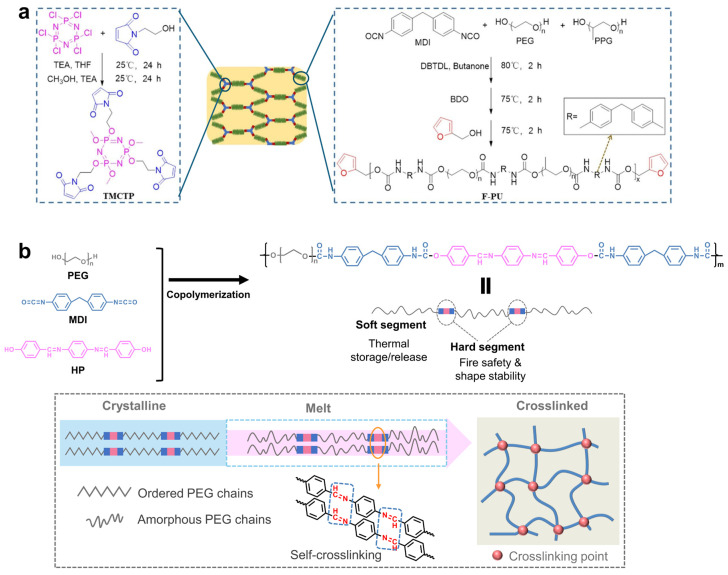
(**a**) Schematic illustration of the synthesis of FPCMs; Reproduced with permission [79]. (**b**) The structure of HPPEG-PU and flame-retardant mechanism. Reproduced with permission [18].

In situ co-polymerization of flame-retardant and phase-change molecules into polyurethane (PU) chains not only mitigates leakage but also enhances the flame retardancy of PCMs. Du et al. [79] synthesized tri-maleimide end-capped cyclotriphosphazene flame retardant (TMCTP), and subsequently fabricated flame-retardant cross-linked PCM composites (FPCMs) by bonding poly(ethylene glycol) (PEG, serves as PCMs) and TMCTP to PU skeleton through a reversible furan/maleimide Diels–Alder (DA) reaction (Figure 3a). The PHRR and THR of the FPCMs decreased from 488.3 to 362.8 W·g^−1^ and from 22.70 to 17.29 kJ·g^−1^, respectively, compared with the PCM without TMCTP. In addition, the char yield of the FPCMs increased to 8.74 wt%, while the LOI value rose to 23.9%. The enhanced flame retardancy of FPCMs originates from the intumescent flame-retardant mechanism associated with P-containing compounds (phosphates and phosphonates) generated during combustion. Moreover, the FPCMs exhibited excellent solid–solid phase change behavior, attributed to the chemical bonding of PEG chains within the crosslinked PU network. Wang’s group [18] innovatively introduced aromatic Schiff base structures and PEG into PU main chains to form solid–solid PCMs (Figure 3b). The aromatic Schiff base units, located within the hard segments of the copolymer matrix, undergo cross-linking reactions at elevated temperatures or during combustion. This cross-linking promotes the formation of an aromatized char layer, which effectively inhibits mass loss and enhances the fire resistance of the PCMs. Moreover, tetrabromobisphenol A (TBBPA) [80], three phosphorus oxychloride (POCl_3_) [81], and phenylphosphonic acid (PPA) [82] were incorporated into the PU skeleton along with PEG segments to develop leak-free, flame-retardant PCMs.

Since solid–liquid PCMs are prone to leakage during phase transitions and are inherently flammable, incorporating them, along with flame-retardant molecules, into a PU skeleton containing chemical and/or physical crosslinking points represents a promising strategy for developing flame-retardant solid–solid PCMs. Analogous to recent developments in thermo-stable polymeric surfactants designed for hot-fluid environments [83], integrating such chemically robust backbones into PCM frameworks could further enhance interfacial stability and thermal endurance.

#### 2.2.3. Other Intrinsic Flame-Retardant PCM Molecules

A series of intrinsic flame-retardant PCMs containing P and Si were prepared by transesterification of diethyl phosphite with 1-tetradecanol, 1-hexadecanol and 1-octadecanol, followed by ring-opening reaction and hydrolytic condensation of 2,3-epoxypropoxy propyltrimethoxysilane (silane coupler KH-560) [84]. The co-incorporated P and Si accelerated the formation of chars and improved the thermal stability and fire resistance of PCMs. Zhao et al. [17] synthesized a series of Schiff base-based PCMs (SB-PCMs) via aldimine condensation of octadecylamine with various aldehydes (benzaldehyde, p-hydroxybenzaldehyde, salicylaldehyde, and 3-hydroxybenzaldehyde). The results demonstrated that the intermolecular hydrogen bond in SB-PCMs played a crucial role in the self-crosslinking of Schiff base groups, which promoted partial charring and imparted corresponding fire-shielding properties. Wang et al. [85] proposed an intrinsic flame-retardant PCM (VOD) that integrates phase change groups with an acid source (diethyl phosphite), a carbon source (vanillin), and a gas source (amines), achieving self-extinguishing within 30 s of ignition. Around the same time, Li et al. [86] developed a similar intrinsic flame-retardant PCM by substituting vanillin with furfural, which resulted in a 73.7% reduction in PHRR compared to pristine octadecylamine. During thermal decomposition, the flame-retardant PCM releases furan, P-, and N-containing fragments. Furan ring degradation yields unsaturated hydrocarbons that promote char formation, while P-containing species convert into oxyacids that catalyze dehydration and carbonization. Through crosslinking and thermal rearrangement, P- and N-rich char layers form, acting as effective barriers that inhibit heat transfer and gas diffusion, thereby enhancing flame retardancy.

Although intrinsic flame-retardant PCM molecules can achieve excellent flame retardancy, their practical applications are still hindered by leakage during phase transitions, which compromises thermal reliability and structural integrity. A promising strategy to address this limitation is to combine these molecules with aerogels, whose highly porous and thermally stable network not only prevents leakage but also contributes synergistically to enhanced flame-retardant performance.

## 3. Aerogel-Based PCMs

**Figure 4:**
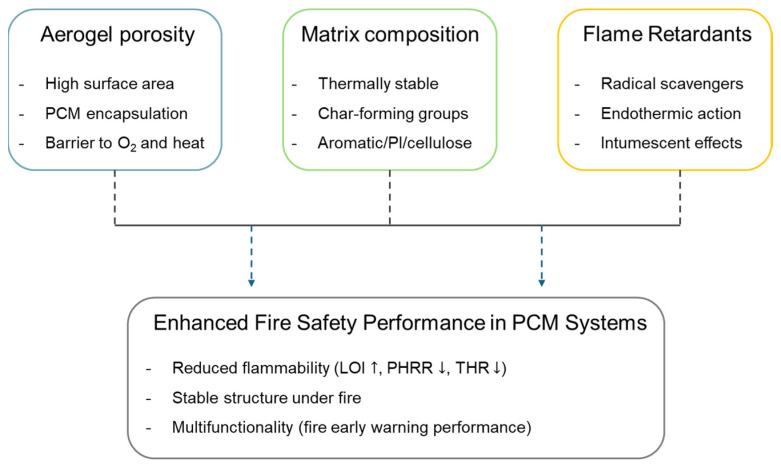
Schematic illustration of the synergistic effects of aerogel porosity, chemical composition, and flame-retardant additives on improving the fire safety of PCM composites.

Aerogels hold significant promise for high-performance composite PCMs owing to their low density, high porosity, large specific surface area, and tunable mechanical properties. The enhanced fire safety of aerogel-based PCMs arises from the synergistic interaction between the aerogel’s porous architecture, its chemical matrix composition, and the incorporation of FR additives (Figure 4). Firstly, the intrinsic porosity of the aerogel network physically encapsulates PCMs, effectively preventing leakage during phase transition and acting as a thermal barrier by limiting heat and oxygen diffusion. Secondly, the chemical structure of the aerogel matrix—such as the presence of thermally stable backbones (e.g., polyimide, silica, or cellulose) and char-forming functional groups—contributes to improved thermal stability and facilitates the formation of protective char layers upon combustion. Thirdly, FR additives (e.g., phosphorus compounds, MXene, metal hydroxides) further enhance flame retardancy through gas-phase radical scavenging, endothermic decomposition, and the generation of intumescent or ceramic barrier layers in the condensed phase. When combined, these three elements operate in concert to reduce flammability, lower PHRR and THR, and increase LOI, resulting in multifunctional energy storage systems that maintain high safety under fire exposure. The classification of aerogels can be divided into three principal categories: inorganic aerogels, organic aerogels, and organic–inorganic composite aerogels.

### 3.1. Inorganic Aerogels

Inorganic aerogels are a class of highly porous, low-density materials typically derived from oxide networks such as silica, alumina, and zirconia. Their unique 3D nanostructure imparts exceptionally high specific surface area, ultralow thermal conductivity, and excellent flame-retardant performance, distinguishing them from conventional porous materials. This method can effectively prevent the leakage of organic PCMs and stabilize their shape in the molten state, but can also generate a high latent-heat capacity due to an extremely high PCM loading in the lightweight porous matrix.

#### 3.1.1. Silica Aerogel-Based PCMs

Silica aerogels (SAs), known for their low density, large specific surface area, excellent thermal insulation and high porosity, show strong potential in various applications such as aerospace, building materials, electronic equipment and other areas [10]. Khedkar et al. [87] tailored the performance of SAs—including surface hydrophobicity, porosity, gel shrinkage, and density—through surface modification using trimethylchlorosilane (TMCS). The flame-retardant effect of pure SA was mainly attributed to its physical barrier function, which provided effective thermal insulation for the underlying material [88,89]. Du et al. [90] demonstrated that incorporating silica-encapsulated n-octadecane (*n*-OD) into aerogels yields phase-change composites with enhanced mechanical strength and reduced thermal conductivity. In addition, the synergistic effects of the silica aerogel skeleton and silica shell not only improve thermal management by delaying temperature rise but also suppress heat release (24.4%) and smoke generation (14.4%), owing both to the inherent flame retardancy of the aerogel and to the dense thermal insulation layer formed by the silica shell, which hinders the release of combustible decomposition products.

Although the SAs discussed above exhibit fire safety, their flame-retardant effectiveness in PCMs remains limited. Consequently, recent studies have explored the bulk incorporation of FRs into PCMs to achieve enhanced fire resistance. By integrating the complementary advantages of SA’s thermal insulation and PCM’s heat dissipation, along with the flame-retardant effect of EG, thermal runaway propagation in battery modules can be effectively mitigated [91]. This strategy provides a practical solution to the long-standing trade-off between insulation and dissipation, offering valuable guidance for the design of next-generation battery thermal management systems.

**Figure 5:**
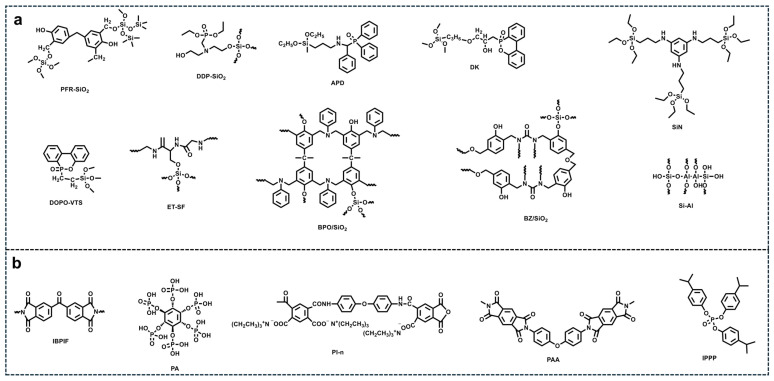
(**a**) Chemical structure of the flame-retardant SA precursors; (**b**) Representative additive flame retardants applied to SAs.

Besides the bulk addition of FRs into PCMs to enhance the flame resistance of SA-based PCMs, direct modification of SAs represents another effective strategy. In situ incorporation of flame-retardant elements such as P, N, benzene rings, and aluminum (Al) into SAs has been shown to further improve their flame-retardant properties; several representative Si-based precursors containing flame-retardant elements are illustrated in Figure 5a [88,92,93,94,95,96,97,98,99,100]. In addition, the use of additive FRs containing P, N elements into SAs has also been reported to enhance their flame resistance (Figure 5b) [101,102,103,104,105,106]. These elements contribute synergistically to both gas-phase and condensed-phase flame-retardant mechanisms. Specifically, P-based compounds promote the formation of char layers, which act as physical barriers against heat and mass transfer, and/or facilitate radical scavenging. N-containing species release non-flammable gases that dilute combustible volatiles. The integration of such FR additives into the porous aerogel network can thus improve the thermal stability, reduce the PHRR, and extend the ignition time of SA-based composites, making them more suitable for fire-sensitive applications. The incorporation of graphene oxide (GO) into SAs can enhance their thermal stability and electrical conductivity [107]. Moreover, coating SAs with functional materials such as organic montmorillonite [108], Mg(OH)_2_ [109,110], BN nanosheets [111], TiO_2_ [112], and ceramic fiber [113] has proven effective in further improving flame retardancy.

#### 3.1.2. Sepiolite Aerogel-Based PCMs

Sepiolite (Sep), with the chemical formula Si_12_Mg_8_O_30_(OH, F)_4_·(H_2_O)_4_. 8H_2_O is a naturally occurring fibrous clay with a layered chain structure that has gained attention as an effective flame-retardant additive for aerogels [114,115]. Jiang et al. [116] synthesized a high-performance sepiolite-based aerogel using freeze-drying technology, in which PA was precisely encapsulated through a vacuum impregnation method to form shape-stabilized mineral composite PCMs (MCPCMs). MCPCMs, with a solidification enthalpy of up to 204 J⋅g^−1^, exhibit self-extinguishing behavior once removed from the fire source. Typically, the char barrier formed by SEP restricts oxygen access to the combustion zone, thereby enhancing the flame retardancy of PCMs.

#### 3.1.3. Carbon Aerogel-Based PCMs

The interconnected network of graphene aerogels (GAs) not only enhances the thermal properties of PCM composites but also facilitates rapid heat dissipation during combustion through their high porosity, thereby imparting excellent fire-retardant performance [117,118,119]. Lin et al. [120] fabricated graphene/boron nitride (GB) aerogels with dual thermal conductivity networks through a self-assembly process involving reduction and calcination. The GB aerogels were subsequently vacuum-impregnated with paraffin wax (PW) to obtain PW-GB composite PCMs. Among them, the PW-G_4_B_2_ PCM exhibited a high phase change enthalpy (135.19 J g^−1^) and excellent shape stability under ignition due to the high thermal stability of BN fibers and the barrier effect of the graphene layers.

Although most inorganic materials exhibit inherently low flammability, their fragility and brittleness must be improved for applications where mechanical strength is critical. Moreover, their complicated preparation procedures and harsh treatment conditions further limit their practical applications.

### 3.2. Organic Aerogels

Organic aerogels are 3D nanoporous materials that offer several advantages over inorganic counterparts, including diverse chemical structures, tunable mechanical strength, and ease of processing. Nevertheless, their inherent flammability significantly restricts practical applications. To overcome this limitation, various strategies have been explored, such as incorporating flame-retardant components into the organic aerogel matrix or employing intrinsically flame-retardant organic precursors. Existing reviews have primarily focused on the processing methods, classifications, physical properties, and flame-retardant mechanisms of organic aerogels [15,121]. Building on the general strategies applied to organic aerogels, Section 3.2.1 will examine recent flame-retardant approaches for organic aerogel-based PCMs, which adopt methods that differ in certain aspects from those used for pure organic aerogels.

#### 3.2.1. Polymer Aerogel-Based PCMs

Polymer aerogels have emerged as promising candidates for energy storage applications due to their ultralow thermal conductivity, low density, high porosity, low dielectric constant, excellent mechanical strength, and tunable molecular structures [122]. However, their inherent flammability and poor thermal stability remain critical limitations [123]. To enhance the performance of polymer aerogel-based PCMs, various strategies have been explored, including physical blending of FRs into PCMs (as discussed in Section 2.1), development of intrinsically flame-retardant PCMs (as discussed in Section 2.2), and in situ incorporation of FR elements into the aerogel matrix.

**Figure 6:**
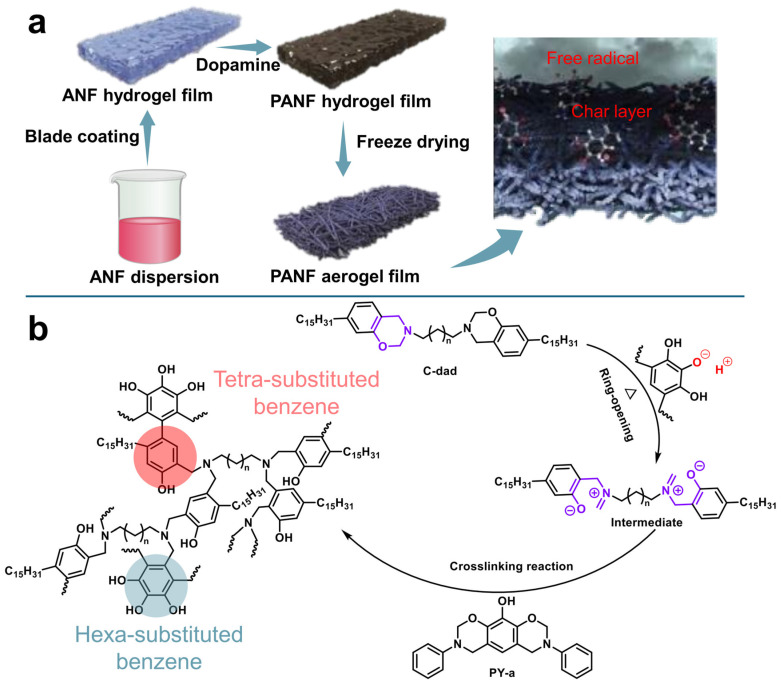
(**a**) Schematic description of the PANF-DES host–guest film and its flame-retarding performances; Reproduced with permission [125]. (**b**) The proposed mechanism of the crosslinking reaction between C-dad and poly(PY-a) [126].

Zhou et al. fabricated APP/BN-functionalized PVA aerogels, into which PEG was incorporated as a PCM via a vacuum-impregnation method, yielding a multifunctional composite PCM (LP-PBAA) [124]. Compared with pure PEG, the maximum decomposition rate and the PHRR of LP-PBAA declined by 47% and 34.1%, respectively. APP can inhibit the pyrolysis of PEG, while the synergistic effect of BN and APP promotes char formation, leading to an increased carbon residue in the PCM composite compared to pure PEG. An organic polydopamine-aramid nanofiber (PANF) aerogel film with a LOI value of 32% was prepared via the in situ polymerization of dopamine within an aramid nanofiber hydrogel matrix (Figure 6a) [125]. The resulting PANF aerogel was subsequently employed as a host to incorporate a ternary deep eutectic solvent (DES), composed of ammonium chloride (NH_4_Cl), ethylene glycol (Eg), and deionized water (H_2_O), which possesses inherent flame-retardant characteristics. The synergistic combination of the flame-retardant aerogel framework and the DES-based PCM endowed the PANF-DES composite PCM with outstanding fire safety features, including reduced heat release, negligible flame propagation, and the absence of visible flames under real fire conditions, thereby ensuring enhanced safety in practical applications. The flame-retardant mechanism primarily arises from the gas-phase radical scavenging capability of catechol functional groups and the ability of polydopamine (PDA) to promote carbonaceous char formation at elevated temperatures.

A simple synthesis strategy for flame-retardant aerogel-based PCMs was developed by physically blending PVA and carboxymethyl cellulose (CMC) as the aerogel framework, *n*-OD as the PCM, and aluminum hydroxide (ATH) as the additive FR, followed by vacuum freeze-drying [127]. The resulting PVA/OD-ATH aerogels demonstrated remarkable improvements in both thermal conductivity and flame retardancy. Liu et al. [126], designed a novel flame-retardant aerogel-based PCM that avoids the use of conventional flame-retardant components by integrating a polybenzoxazine-based aerogel (PB-1) with benzoxazine-based PCMs (C-dad). The flame-retardant mechanism is shown in Figure 6b. At elevated temperatures, phenolic hydroxyl groups on the aerogel skeleton initiate the cationic ring-opening polymerization (ROP) of C-dad monomers, forming zwitterionic intermediates. These intermediates undergo two main reaction pathways, leading to hexa- and tetrasubstituted benzene linkages, resulting in a crosslinked polybenzoxazine copolymer monolith. This network alters the pyrolysis behavior of the original phase change composite, enhancing its charring ability and overall flame resistance. This unique structural integration enables the obtained composite (PB-1/C-dad) to achieve a latent heat of 145.3 J g^−1^, increased char yield of 13.1% at 600 °C, and a PHRR of 231 W g^−1^, surpassing many representative flame-retardant polymer/organic PCM hybrids reported in the literature. Recent studies on thermally resilient polymeric frameworks under dual-scale boundary conditions, such as sodium p-styrenesulfonate modified polyacrylamide systems, further highlight the importance of interfacial polymer design for maintaining stability under thermal stress [128]. In addition, metal–organic framework (MOF)–aerogels offer significant advantages for energy storage applications due to their enhanced structural stability, tunable functionality, and high energy storage efficiency [129].

#### 3.2.2. Biomass Aerogel-Based PCMs

Derived from natural structures, bio-based materials can be obtained through a facile “top-down” approach, avoiding the need for complex fabrication processes. Among them, cellulose and its derivatives—polysaccharides that represent the most abundant natural polymers on Earth—are particularly attractive due to their renewability, biocompatibility, and biodegradability. A CMC/benzoxazine-olefin copolymer (AMBZ) composite aerogel was prepared by phosphoric acid-catalyzed ROP of AMBZ with CMC, followed by directional freezing and freeze-drying [130]. PEG was then encapsulated within the composite aerogel to produce composite PCMs, which exhibited a significant decreased initial thermal decomposition temperature due to the presence of unstable O-P-O bonds in phosphoric acid. However, the crosslinked polyphenyloxazine structure in the aerogel contributed to the higher *T*_max_ (the temperature corresponding to the peak mass loss rate).

**Figure 7:**
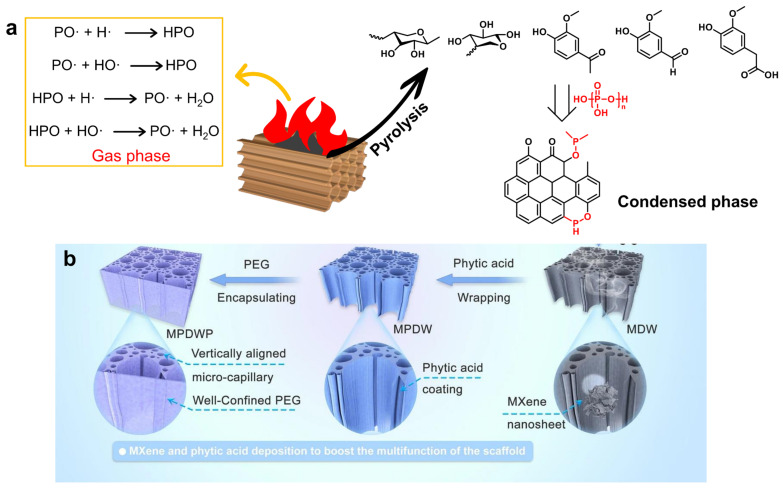
(**a**) The flame-retardant mechanism of Delig-wood/PA aerogel. (**b**) The preparation process of composite phase change materials supported by phytic acid and MXene-decorated wood aerogel. Reproduced with permission [134].

The hierarchical porosity (macro- to nanoscale) of wood makes it a promising functional material, and its fire resistance can be enhanced through delignification and densification [131] as well as co-impregnation with multicomponent FRs [132]. For example, Delig-wood/PA aerogel was fabricated by partially delignifying natural wood using a NaClO_2_/acetic acid solution, followed by doping with phytic acid (PA) and MXene via an evaporation-induced self-assembly method [133]. During combustion, free radicals such as PO· generated from the pyrolysis of PA effectively scavenged active radicals, including H· and HO·. Simultaneously, the pyrolysis products of wood and PA underwent crosslinking reactions, leading to the formation of a stable residue enriched in C-O-P and C=C structures (Figure 7a). This residue contributed to combustion suppression, enabling the aerogel to achieve a V-0 rating in the UL-94 test. Chen et al. [134] prepared composite PCMs using the above-mentioned wood aerogels as the supporting skeleton and PEG as the PCM (Figure 7b), achieving leakage-free PEG encapsulation while simultaneously enhancing thermal conductivity, light absorption, and flame retardancy. MXene/PA hybrid modification preserved the open-pore structure of the nanowood scaffold, leaving the PEG encapsulation capacity unchanged. Moreover, the increased exposure of hydrophilic functional groups (e.g., –OH, –COOH, and –F) enhanced hydrogen bonding interactions with PEG, contributing to improved structural compatibility and stability. The interaction of decomposition gases with P-containing groups, together with the formation of protective char layers catalyzed by MXene nanosheets, synergistically contributed to the enhanced flame-retardant performance of the wood-derived composite PCMs. Yue et al. [135] designed Ti_3_C_2_T_x_ MXene/delignified wood aerogels deposited with ammonium dihydrogen phosphate (ADP), which served as supporting matrices for *n*-OD, resulting in flame-retardant, form-stable PCMs (PMPCMs). The composites exhibited a reduction in PHRR from 1579.6 to 976.2 W g^−1^ and a decrease in THR from 48.6 to 38.4 kJ g^−1^. The thermal decomposition of ADP generated P-containing products (e.g., HPO_3_) that reacted with cellulose, promoting dehydration and the formation of a P-rich carbonaceous layer with excellent fire-protective performance [136]. Wang et al. synthesized a phosphorus/ammonium-containing, formaldehyde-free flame retardant (APA) based on PA, and grafted it onto delignified wood to produce flame-retardant wood aerogels [137]. The form-stable PCM composites (PTPCMs) were then obtained by impregnating the wood aerogel with a thermochromic system of myristyl myristate (C_28_H_56_O_2_) and methyl red (C_15_H_15_N_3_O_2_), and they exhibited high energy-storage density (165.3–198.6 J g^−1^), reduced PHRR and THR, and real-time, visually reversible thermochromism. A potential flame-retardant mechanism was proposed: upon heating or combustion, the aerogel skeleton decomposes and releases a significant amount of nonflammable gases such as CO_2_, H_2_O, and P–O species. These inert gases absorb heat and dilute both flammable volatiles and ambient O_2_, thereby suppressing combustion. Simultaneously, the decomposition of APA contributes to the formation of compact, continuous, and expanded char layers, which serve as protective barriers. These layers reduce smoke emission and carbon monoxide production, thereby enhancing the thermal stability and overall fire safety of the PCM composite.

### 3.3. Organic–Inorganic Aerogel-Based PCMs

One advantage of inorganic aerogels lies in their intrinsic fire resistance; however, they are often limited by brittleness and poor mechanical strength, particularly in the case of inorganic silica SAs [138]. By contrast, organic aerogels typically offer greater flexibility but suffer from high flammability and thermal instability [15]. To reconcile this trade-off, organic–inorganic hybridization provides a promising strategy to combine their strengths while mitigating their respective shortcomings. Incorporating inorganic fillers such as carbon nanotubes (CNTs), graphene, silica, and metal oxides or hydroxides (e.g., ZnO or Al(OH)_3_) has proven effective in enhancing both the flame retardancy and other functional properties (e.g., thermal conductivity) of aerogels and their associated PCMs, as shown in Table 2.

**Table 2:** Presentative aerogel-based flame-retardant PCMs.

Aerogels	PCMs	FRs	Δ*H*_m_ (J g^−1^)	PHRR	THR	Ref.
Surface-carbonized delignified wood	*n*-Docosane	Phytic acid/zinc oxide (ZnO)	185.2	592.1/737.4 W g^−1^	43.5 KJ g^−1^	[139]
Delignified wood	*n*-Docosane	Phytic acid/melamine (MEL)-modified Nb_2_CT_x_ MXene	190.2	532.3/589.4 W g^−1^	39.7 KJ g^−1^	[140]
Wood powder/CNT/calcium alginate	PEG	-	132.4	36.0 W g^−1^	-	[141]
BN/Co-MOF	PA	-	188.1	-	-	[142]
Polyimide (PI)/sodium lignosulfonate-zirconium phosphate (SL-ZrP)	PEG	-	152.8	667.0 W g^−1^	23.3 KJ g^−1^	[143]
KF/SA/MXene	PEG	Phytic acid	110.4	613.0 kW m^−2^	170.7 MJ m^−2^	[144]
MXene/polyimide (PI)	PEG	-	167.9	529.3 W g^−1^	21.4 KJ g^−1^	[145]
Alginate/phytate/BN	Melamine resin@Aliphatic alcohol microcapsule	-	-	48.4 kW m^−2^	6.4 MJ m^−2^	[146]
Graphene-modified PVA	PEG	-	164.2	448.7 W g^−1^	22.2 KJ g^−1^	[147]
PVA/CMC	Octadecane	Aluminum hydroxide	134.6	293.9 W g^−1^	38.8 KJ g^−1^	[127]
SA	Octadecane	Silicon dioxide	114.1	197.9 kW m^−2^	17.0 MJ m^−2^	[90]
BN	PA	-	183	-	-	[148]
Graphene	Paraffin wax	-	141.9	481.7 W g^−1^	44.6 MJ m^−2^	[149]
SA	PA	PU/APP/DEPR coating	79.2	-	-	[19]
PI/BC/SiO_2_	*n*-OD	-	232.5	1007.8 W g^−1^	44.5 KJ g^−1^	[150]
PVA/CNT@TA	P-modified PEG	-	131.9	220.3 kW m^−2^	131.1 MJ m^−2^	[151]

Note: Units are specified individually to reflect the original measurement technique. kW·m^−2^ values are obtained from cone calorimeter tests, while W·g^−1^ values are from microscale combustion calorimetry (MCC).

**Figure 8:**
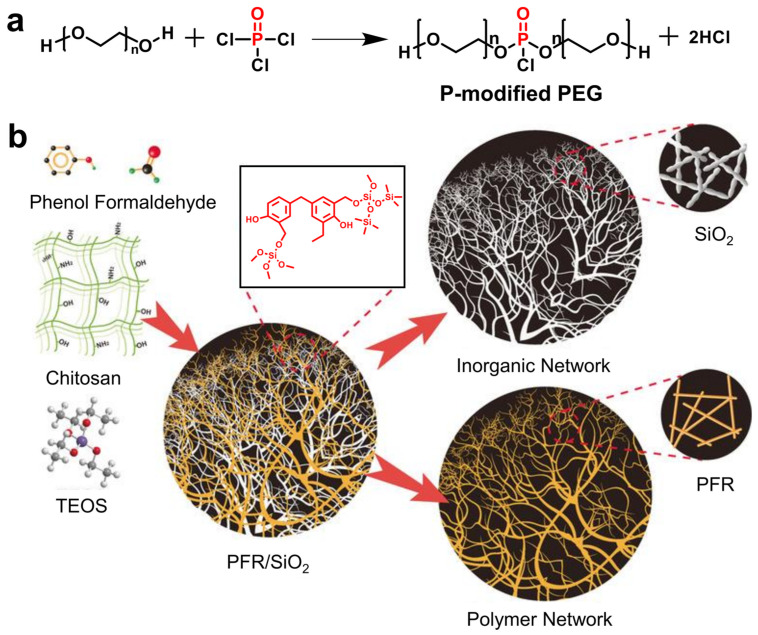
(**a**) The preparation of P-modified PEG. (**b**) Schematic illustration of the synthesis and structural composition of PFR/SiO_2_ composite aerogel with an interpenetrating binary network [93].

The polyimide (PI)-hydroxyapatite-reduced graphene oxide (PI-HAP-rGO) aerogel fabric (outer layer) achieved reductions in PHRR of 54.7% and 70.0% compared with pure PI aerogel fabric and commercial aramid fabric, respectively, demonstrating outstanding fire resistance [152]. In addition, zeolitic imidazolate framework-8 (ZIF-8) was reported as a flame retardant in PVA aerogels by increasing the residue yield [153]. Recent progress in interpenetrating double-network ANF/MXene aerogels demonstrates the potential for multifunctional integration of thermal management, electromagnetic shielding, and Joule heating, providing inspiration for designing next-generation flame-retardant PCM aerogels with coupled energy and safety functionalities [154]. Inspired by the natural porous structure of coral, a coral-like organic–inorganic graphene-modified PVA aerogel (GP) was developed as a host for PEG, yielding a composite PCM (P-GP) with enhanced thermal conductivity (0.7545 W·m^−1^·K^−1^) and reduced PHRR and THR owing to the incorporation of a nonflammable graphene network [147]. Long et al. [155] developed a PVA/amino-BN hybrid aerogel as a supporting skeleton for flame-retardant P-containing PEG (P-PEG). P-PEG was prepared by phosphorylating PEG with POCl_3_ to form phosphate ester (PEG-O-P=O) linkages, with HCl as the byproduct, as shown in Figure 8a. The combination of P-PEG and amino-BN markedly promoted carbon formation, thereby enhancing flame retardancy, as reflected by an 84.7% reduction in PHRR and a 62.8% reduction in THR. Shen et al. [156] reported that PEG-LBN@PI composites, based on lignin-modified BN/PI hybrid aerogels, achieved 23.3% and 17.3% reductions in PHRR and THR, respectively, compared with pure PEG. LBN@PI imparts excellent thermal stability to PEG composites, with increasing PI content resulting in greater char residue and enhanced fire resistance. Two-dimensional (2D) layered black phosphorus (BP) was synthesized from bulk BP crystals via ultrasonication-assisted liquid exfoliation and subsequently employed as an inorganic flame-retardant additive to construct cellulose nanofiber (CNF)/BP hybrid aerogels [157]. These hybrid aerogels were then impregnated with n-octacosane to fabricate fire-safe, form-stable PCMs (CBPCMs). The incorporation of BP facilitated the formation of an expanded and continuous honeycomb-like char residue, indicative of strong intumescent char development during combustion. As a result, both the PHRR and THR were significantly reduced, while the LOI of the composites was notably enhanced.

However, conventional inorganic fillers face limitations such as poor compatibility with polymer matrices, which can lead to disintegration during combustion and insufficient protection against thermal structural collapse. To overcome this challenge, phenol-formaldehyde-resin (PFR)/SiO_2_ composite aerogels were fabricated through a direct co-polymerization and nanoscale phase-separation strategy [93]. Phenol and formaldehyde served as organic monomers, while tetraethyl orthosilicate (TEOS) acted as the inorganic precursor (Figure 8b). The resulting interpenetrating binary network effectively mitigates incompatibility between the components, yielding aerogels with enhanced mechanical robustness and superior fire-retardant performance. When it was exposed to a propane/butane blowtorch flame, the carbonized backside and the silica shell on the front side remained firmly integrated, effectively preventing structural collapse. This enables the aerogels to withstand prolonged exposure to high-temperature flames without undergoing structural collapse or catastrophic failure.

**Figure 9:**
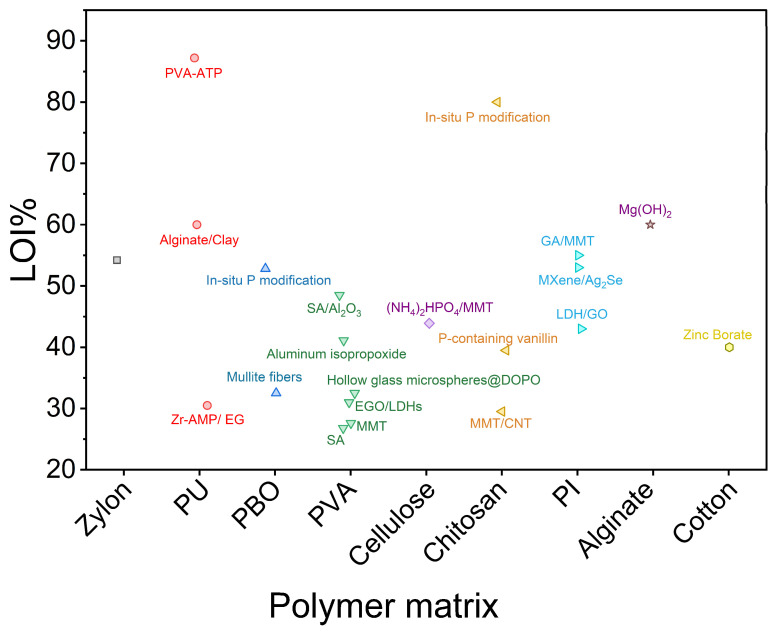
Comparative bar chart of limiting oxygen index (LOI) values for organic–inorganic aerogels. Composite systems are grouped by aerogel matrix type, including PU, PVA, polyimide (PI), chitosan, and others.

In addition to the structural advantages provided by aerogel frameworks, the flame-retardant mechanisms within organic–inorganic aerogel-based PCM composites are highly dependent on the interaction between the aerogel matrix and the embedded additives. In the condensed phase, materials such as layered silicates, polyphosphates, and MXenes promote the formation of a thermally stable char layer, which acts as a physical barrier to heat and oxygen diffusion. The high porosity and specific surface area of the aerogel scaffold enhance this effect by facilitating localized thermal degradation and carbonization, thus accelerating char yield and improving its structural coherence. Simultaneously, gas-phase mechanisms—notably radical scavenging—are often enabled by functional inorganic fillers (e.g., metal oxides, LDHs, aluminum hypophosphite), which release water, CO_2_, or inert gases upon decomposition, diluting combustible volatiles and interrupting flame-propagating radicals (·H, ·OH, ·O). These mechanisms are further strengthened by the nano-confinement within aerogel pores, which can delay volatile release and stabilize thermal decomposition. As a result, aerogel-based composites exhibit synergistic flame retardancy by coupling physical insulation with chemical reactivity, which is not typically achievable in conventional PCM matrices. To further clarify the relationship between aerogel matrix types and flame-retardant performance, Figure 9 presents a comparative analysis of LOI values across various organic–inorganic aerogels. The figure highlights the effect of polymer matrix and inorganic components loading on LOI performance, illustrating that systems based on PVA and chitosan generally achieve lower LOI values, indicating lower flame retardancy. The introduction of P elements in aerogels can efficiently improve flame retardancy. Data are sourced from the literature [158,159,160,161,162,163,164,165,166,167,168,169,170,171,172,173,174,175,176,177,178].

## 4. Conclusions and Prospects

Flame-retardant aerogel-based PCMs represent a rapidly advancing class of functional composites that address two critical limitations of traditional PCMs: leakage during phase transition and high flammability. Aerogels, with their ultralow density, high porosity, and tunable microstructures, provide an effective scaffold for PCM encapsulation, offering improved shape stability, mechanical strength, and thermal insulation. Simultaneously, integrating flame-retardant strategies—ranging from physical blending of additives to intrinsic chemical modification—has proven effective in enhancing fire safety through radical quenching, char formation, and barrier effects. This review has provided a comprehensive overview of the recent advancements in aerogel-based PCMs with integrated flame-retardant properties. By classifying materials based on their chemical nature and flame-retardant mechanisms, and by examining the interplay between structure, composition, and multifunctional performance, we offer a novel perspective that bridges the gap between energy storage and fire safety. Unlike prior reviews that treated these aspects separately, this work emphasizes the necessity of coupling energy storage/release with fire resistance in the practical deployment of PCMs. However, there are still several issues that need to be further studied and addressed.

(1) Recycled aerogels and flame retardants for sustainable PCM composites

Future research should prioritize the use of recycled or waste-derived resources for aerogel matrices. Industrial by-products (e.g., fly ash, lignocellulosic residues) and polymer waste streams can be re-engineered into aerogel frameworks, thereby reducing environmental impact while lowering production costs. For example, Do et al. [179] converted 100% of fly ash into a lightweight composite aerogel reinforced with recycled polyethylene terephthalate fiber. Moreover, the low durability and complex composition of conventional FRs pose both economic and environmental challenges for flame-retardant PCMs. Leaching and reduced efficiency shorten service life, while discarded FRs lead to global pollution concerns. Wang’s group [180] designed microcage structures with infinite chemical recyclability to the original monomers, exceptional durability, and versatile flame-retardant performance through the hierarchical assembly of phosphoric acid (PA) and Cu^2+^ monomers. The dynamically reversible covalent network within the microcages can be readily disassembled by environmentally friendly H^+^ triggers, enabling efficient chemical recycling with high yields—92.0% for PA and 96.2% for Cu^2+^-based monomers. Future development should focus on durable, recyclable, and eco-friendly systems—such as bio-based additives, stable chemical integration into aerogel matrices, and circular recycling strategies—to balance performance, cost, and sustainability.

(2) Biodegradable aerogels and flame retardants

Biodegradable FRs offer a promising route to reconcile fire safety with environmental sustainability. Unlike conventional additives that persist and accumulate in ecosystems [181], biodegradable systems—derived from natural polymers or functionalized with eco-friendly phosphorus, nitrogen, or silicon groups—can degrade into non-toxic products after use. Some reviews summarized the use of biodegradable and bio-based compounds for flame retardants used in polymeric materials [182]. The challenge lies in achieving sufficient durability and flame-retardant efficiency during service while ensuring controlled degradation at end-of-life. Future efforts should focus on designing multifunctional biodegradable systems that combine fire resistance with structural stability, while integrating lifecycle assessment and green synthesis to guide their development. For example, Karatum et al. [183] conducted a cradle-to-grave streamlined life cycle assessment (LCA) of scaled, commercially viable fabrication methods for polyurethane foam (PUF) and Aspen Aerogels to evaluate their environmental impacts and determine their suitability for deployment in oil spill response scenarios. Zhang et al. [184] synthesized an efficient and environmentally friendly N–P synergistic flame retardant, APGDPE, under solvent-free conditions using non-toxic reagents—1,3-propylene glycol and phosphoric acid—as reactants. Aerogels, particularly those derived from natural polymers such as cellulose, chitosan, starch, or lignin, hold great promise for eco-friendly PCM systems. These bio-aerogels combine renewability with tunable porosity, providing efficient PCM encapsulation. Incorporating biodegradable FRs into such biodegradable matrices could balance energy storage, fire safety, and environmental compatibility, enabling applications in packaging, building insulation, and transient electronics.

(3) Machine learning-driven design and optimization

Current attempts rely only on an experimental approach, which has inherent shortcomings, including inefficiency due to the prolonged synthesis process, and the necessity of analyzing microstructure and properties. In order to address the challenges associated with the traditional experimental approach. Machine learning (ML) is expected to play an increasingly important role in the design and optimization of flame-retardant aerogel-based PCMs. For example, Zhang et al. [185] predict the flame retardancy index for different types of flame-retardant polymeric nanocomposites using five machine learning algorithms, and then designed and synthesized a high-performance flame-retardant polymeric nanocomposite. Tafreshi et al. [186] predicted various material properties of PI aerogels, including compressive modulus, density, and porosity, using an artificial neural network (ANN). Given the complex interplay between material composition, microstructure, thermal energy storage capacity, and flame retardancy, ML algorithms can also be employed to model structure–property relationships of aerogel-based PCMs and predict performance outcomes based on large experimental or simulated datasets. Supervised learning models, for example, could assist in optimizing PCM loading, aerogel porosity, and flame-retardant additive ratios to achieve desired thermal and fire-safety performance. Moreover, ML could facilitate the identification of novel formulations by screening vast chemical spaces more efficiently than traditional trial-and-error approaches. Although current applications of ML in this specific domain are still limited, its integration with high-throughput synthesis, multi-scale simulations, and data-driven material informatics holds significant promise for accelerating the development of next-generation, multifunctional aerogel-based PCM systems.

(4) Long-term durability and multifunctionality

Despite recent progress, flame-retardant aerogel-based PCM systems still face challenges related to long-term thermal reliability, interfacial degradation, and scalability. Developing thermally and chemically resilient polymer frameworks is essential. For example, thermo-stable polymeric surfactants have shown improved interfacial stability under thermal cycling, offering potential for more durable PCM matrices. Looking forward, multifunctionality will be crucial. Recent advances in ANF/MXene double-network aerogels demonstrate the integration of thermal management, Joule heating, and EMI shielding in a single framework, providing a promising direction for future PCM systems with enhanced safety and adaptability [154]. Future work should aim to combine such capabilities with scalable, sustainable fabrication to enable practical deployment in energy storage applications.

Overall, the development of flame-retardant aerogel-based PCMs holds great potential to advance safe, efficient, and sustainable thermal energy storage solutions. Continued efforts at the intersection of materials innovation, green chemistry, and intelligent design are expected to drive the next generation of high-performance composites for applications in renewable energy, building insulation, and beyond. Despite providing a comprehensive overview of flame-retardant aerogel-based PCMs, this review has several limitations that should be acknowledged. Due to the diversity in material compositions, fabrication techniques, testing standards, and evaluation conditions reported across studies, it is challenging to perform direct quantitative comparisons or establish universally applicable structure–property relationships. While the review highlights a range of flame-retardant strategies, the long-term reliability, recyclability, and environmental impacts of many reported systems remain insufficiently addressed in the literature. Lastly, the scalability of these advanced aerogel-based PCM systems for industrial applications has not yet been thoroughly demonstrated and requires further investigation. Future work should aim to develop standardized testing protocols and assess real-world performance to bridge the gap between laboratory research and practical implementation.

## Data Availability

No new data were created or analyzed in this study.
